# Knowledge, attitudes and practices related to cystic echinococcosis endemicity in Pakistan

**DOI:** 10.1186/s40249-017-0383-2

**Published:** 2018-01-22

**Authors:** Aisha Khan, Kashf Naz, Haroon Ahmed, Sami Simsek, Muhammad Sohail Afzal, Waseem Haider, Sheikh Saeed Ahmad, Sumaira Farrakh, Wu Weiping, Guan Yayi

**Affiliations:** 1 Department of Biosciences, COMSATS Institute of Information Technology (CIIT), Park Road, Chakh Shahzad, Islamabad, Pakistan; 2 Department of Parasitology, Faculty of Veterinary Medicine, University of Firat, 23119 Elazig, Turkey; 3 Department of Life Sciences, University of Management and Technology (UMT), Lahore, Pakistan; 4 Department of Echinococcosis, National Institute of Parasitic Diseases, Chinese Center for Disease Control and Prevention, Key Laboratory of Parasite and Vector Biology, Ministry of Health, WHO Collaborating Center of Tropical Diseases, National Center for International Researches on Tropical Diseases, Ministry of Science and Technology, 207 Ruijin Er Road, Shanghai, 200025 China; 5 Center for Global Health, National Institute of Parasitic Diseases, Chinese Center for Disease Control and Prevention, Key Laboratory of Parasite and Vector Biology, Ministry of Health, WHO Collaborating Center of Tropical Diseases, National Center for International Researches on Tropical Diseases, Ministry of Science and Technology, 207 Ruijin Er Road, Shanghai, 200025 China; 6 Department of Environmental Sciences, Fatima Jinnah Women University, Rawalpindi, Pakistan

**Keywords:** Cystic Echinococcosis, People, Knowledge, Awareness, Practice, Risk, Pakistan

## Abstract

**Background:**

Cystic echinococcosis (CE) is a human and animal health problem in many endemic areas worldwide. It is considered a neglected zoonotic disease caused by the larval form (hydatid cyst) of *Echinococcus* spp*.* tapeworm. There are limited studies on echinococcosis in Pakistan.

**Methods:**

A cross-sectional survey was conducted to find out recent knowledge, attitudes and practices on the occurrence of cystic echinococcosis in butchers and dog owners in both urban and rural areas of Rawalpindi/Islamabad regions, Pakistan. The quantitative data was collected in the form of questionnaires to investigate the knowledge and awareness of CE among community members and their routine practices that  were behind the factors involved in hydatid cyst infection. The practices and infrastructure of abattoirs/butcher shops and their role in transmission of cystic echinococcosis were also evaluated in the present study.

**Results:**

The participants involved in the study were dog owners and people who kept animals. A total of 400 people were interviewed and 289 questionnaires were received**.** The results showed that only 4.1% of people have heard about the disease, and 58.1% were closely associated with dogs. Sixty-three percent of dogs in study area were consuming uncooked organs (e.g. liver, lung, etc.) of slaughtered animals, while 100% of dogs at butcher shops were consuming uncooked organs. Home slaughtering was common in 20.06%. Among butchers, 32.3% had heard about zoonoses and 7.61% knew about CE. The statistical analysis showed that there was highly significant difference (*P* < 0.05) among most of the practices that were associated with the prevalence of CE.

**Conclusions:**

It  was concluded from the present study that, the knowledge and awareness of CE among people of Rawalpindi/Islamabad were low. Because of dogs and poor knowledge of CE among community members and butchers, the transmission of echinococcosis is facilitated. Therefore, there is urgent need to strengthen awareness and health education among people, as well as proper practices related to the CE not only in the study area, but also in other areas of Pakistan.

**Electronic supplementary material:**

The online version of this article (10.1186/s40249-017-0383-2) contains supplementary material, which is available to authorized users.

## Multilingual abstracts

Please see Additional file 1 for translations of the abstract into the six official working languages of the United Nations

## Background

Cystic echinococcosis (CE) is a larval stage disease of small taeniid type tapeworm (*Echinococcus granulosus*) that may cause infection in herbivorous animals and humans. Echinococcosis is one of the 17 neglected tropical diseases (NTDs) stated by the World Health Organization. *E. granulosus* is responsible for causing CE, which affects more than 1 million people around the world and responsible for over $3 billion in expenses every year [1]. The disease has about 1/100 000 prevalence in developed countries, whereas the rate is 10% in developing countries. Approximately 2 – 3 million human cases are thought to occur worldwide [2].

In Central Asia, echinococcosis is endemic and causes serious health problems. Various *Echinococcus* species reside in domesticated or wild mammals. Domesticated dogs and wild carnivores such as foxes, coyotes and wolves may act as definitive hosts, and livestock and humans act as intermediate hosts [3]. Humans become infected through accidental ingestion of food, vegetables, fruits or drinking water contaminated with the eggs of *E. granulosus.* Another possibility of acquiring infection is a direct contact with infected definitive hosts of the parasite [3]. In typical life cycle of *E. granulosus,* adult tapeworms that are usually 3-6 mm long reside in the small intestine of definitive hosts, then hydatid cyst stages occur in herbivorous intermediate hosts, such as sheep, cattle, goats, camels, horses, pigs and humans as well. In a typical dog-sheep cycle, tapeworm eggs are passed in the feces of an infected dog and may subsequently be ingested by grazing sheep; they hatch into embryos in intestine, penetrate intestinal lining, and are then picked up and carried by blood throughout the body to major filtering organs (mainly liver and/or lungs). After localization of developing embryos in a specific organ or site, they transform and develop into larval echinococcal cysts in which numerous tiny tapeworm heads called protoscolices are produced via asexual reproduction. A single cyst can have thousands of protoscolices, and each protoscolex is capable of developing into an adult worm if ingested by the definitive host [4].

There are many social reasons favouring the life cycle of *E. granulosus* and prevalence of CE in various parts of the world. Many families in rural have small plots of land and live in close proximity with their flocks and dogs. The gathering and grazing together of groups of animals belonging to different owners lead to circulation of infections, including CE. Home slaughter and feeding of dogs with raw offals favour the parasite’s life cycle [5]. Various small and poor equipped slaughterhouses built in the area of human settlements, lack of public health education are other factors that favour the life cycle of *E. granulosus*. Stray dogs and other canids, especially wolves may feed on dead animals and garbage, and hunt intermediate hosts. Dogs and livestock living in close proximity with man leads to circulation of zoonotic infection. Moreover, high cost and difficulties of slaughtering single animals consequent to legislative rules may create situations of uncontrolled slaughtering [6].

Since there was little knowledge, attitudes and practices (KAP) studies in Pakistan, the objectives of this study was to determine the KAP associated with CE in a selected study area, and to provide some information for the development of intervention strategies and measures for CE in Pakistan.

## Methods

### Study area

**Fig. 1:**
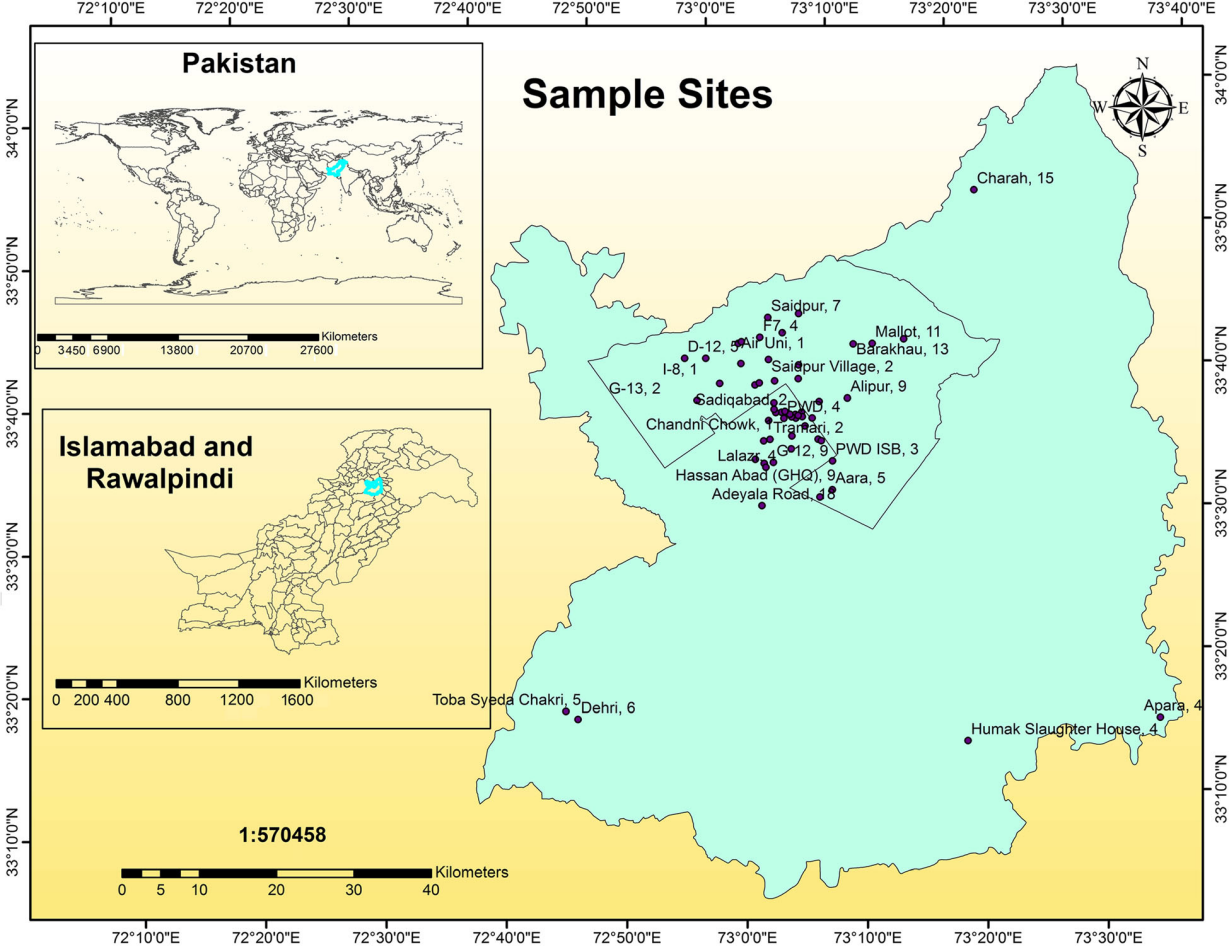
Map of Pakistan showing the location of the study area

The area selected for this survey included the region covering both twin cities of Islamabad and Rawalpindi, while samples were selected from various representative locations (Fig. 1). Islamabad is located at 33.43°N 73.04°E at the northern edge of the Pothohar Plateau and at the foot of the Margalla Hills in Islamabad (Capital City of Pakistan). Its elevation is 540 meters (1 770 ft.) and it is made up of 505 km^2^ of urban land and 401 km^2^ of rural land [7]. The modern capital Islamabad and the city of Rawalpindi stand side by side and are commonly referred to as the twin cities, where no exact boundary exists between the two cities. Rawalpindi and Islamabad regions contain both urban and rural areas. According to census 2017, 47.05% of the population of Rawalpindi and 49.15% of the population of Islamabad belong to rural area. Moreover, 210 hydatid cyst cases have been reported in the study area in the past few years (unpublished data). Eighty-two percent of the population are Punjabi people, 10.3% consist of Pashto people and 7.6% are others. It has a total area of 259 km^2^ (100 sq. mi) and an elevation of about 508 m (1 667 ft.) [8].

### Study duration

The duration of the study was 6 months from January to July 2017. During this period, different abattoirs, butcher shops and villages of Rawalpindi and Islamabad were visited for collection of data concerning prevalence of hydatid cyst in lungs and liver of slaughtered animals. The sub-sampling method was used in this purpose.

### Study design

The study design was a cross-sectional survey that was conducted in two selected cities. The study was carried out in two steps: at first, a door-to-door census of entire population of each area; and secondly, a survey using a structured questionnaire carried out by trained field workers or health workers. The survey was directed to all family members above 15 years old of households including butchers, urban people and villagers in a subset of randomly selected houses.

The study was carried out according to the Declaration of Helsinki Principles, International Conference on Harmonization, and all Pakistan pertinent regulations. Written informed consent was obtained from each participant at enrolment.

To find out recent information on the occurrence of hydatid cyst from different abattoirs, butcher shop and villages of twin cities were analysed. Questionnaire was descriptive in nature, and was designed for butchers (who used to feed dogs with slaughter organs/who had no stray dogs on shops), urban people (who kept dogs) and villagers (who kept dogs and livestock animals). Both qualitative and quantitative data were collected to check the awareness and knowledge of CE among the population and their routine practices that are behind the factors involved in high prevalence rate of hydatid cyst. A total of 289 questionnaires were filled from the twin cities. Data were collected from areas where expected exposure factors such as butchers, people with animals and dogs, and those who had close association with animals were present.

### KAP and socio-demographic characteristics assessment

The questionnaire had a total of 23 questions divided as follows: 5 questions on knowledge of echinococcosis; 2 on each point such as symptoms, treatment, diagnosis, and measures; 7 questions on awareness towards transmission, prevention and diagnosis; and 9 questions on practices such as washing hand before eating food, playing with dogs, etc. For the assessment of socio-demographic characteristics, 5 questions on socio-demographic characteristics were included.

### Inclusion and exclusion criteria

All dog owners (common people and butchers) who were 15 years of age and above with livestock who also possess dogs, were included in the study by selecting one family member from each family. Children less than 15 years of age and houses with livestock but no dogs were excluded from this study.

### Data collection methods

The questionnaires were designed to collect data on socio-demographic characteristics as well as knowledge, awareness, practice related to CE. Moreover, data were analysed to determine the factors associated with knowledge, attitude and awareness towards CE. Since, the disease does not have a specific local name, pictures of infected human and cysts in animal organs were used to explain to the participants. Individuals were asked whether they know the disease or not, hence this was a Yes or No question which was binary. Knowledge and attitude were measured as binary outcomes [9].

### Data analysis

Data were entered into MS Excel spread sheet and a database was established. Statistical analysis was performed using R Statistical Software Version 3.3.0. Chi-square at 95% (CI) was used to examine the factors involved in prevalence of hydatid cyst [10]. The relationship between different factors influencing knowledge, attitudes and practices were analysed. Statistically significant difference was considered if the test results were in *P* < 0.05.

### Study variables

Both independent and dependent variables were included in the study [9].

### Dependent variables

1. Practices/factors associated with spread of CE
2. Knowledge about CE
3. Attitude towards infection with CE

### Independent variables in the study

**Table:** 

S. No	Variable	S. No	Variable
1	Gender of study participants	11	Deworming of dogs
2	Age	12	Home slaughtering
3	Occupation	13	Meat inspection
4	Level of education	14	Feeding of dogs with cysts
5	Animal keeping	15	Handling of dog fecal matter
6	Animal management system	16	Vegetable (raw) consumption
7	Dog ownership	17	Hand washing
8	Using dogs to guard livestock	18	Water treatment
9	Dog confinement	19	Source of water
10	Interaction of human with dogs	20	Waste disposal system

## Results

### Socio-demographic characteristics

A total of 289 questionnaires were filled from three categories i.e. rural (*n* = 99), urban population (*n* = 85), and butchers (*n* = 105), of Rawalpindi & Islamabad. The percentage of questionnaires filled from villagers was 34.3% (99/289) and from urban populations who have dogs was 29.4% (85/289), while the percentage from the sites of butchers and abattoirs was 36.3% (105/289). Participants involved in this study were dog owners and peoples who kept animals. By combining the data from three different sites we concluded that most of the participants were male with 90.3% (261/289), whereas among this value of 90.3% (261/289), 105 butchers were male. The percentage of female participants was low as 9.7% (28/289). The interviewed participants were adults of age group of 15 – 35 years old with highest percentage of 56.7% (164/289) while other age groups are of 36 – 56 years old with 36.3% (105/289) and from 57 – 77 years old with 6.9% (20/289). Furthermost participants were male (Table 1).

**Table 1:** Sociodemographic background of the participants

Variable	Characteristics	No. of Participants *n* = 289	Frequency (%)
Gender	Male	261	90.3
	Female	28	9.6
Age	15 – 35	164	56.7
	36 – 56	105	36.3
	57 – 77	20	6.9
Ethnicity	Punjabi	237	82.0
	Pathan	30	10.3
	Urdu speaking	5	1.7
	Sindhi	3	1.0
	Siraiki	2	0.69
	Kashmiri	4	1.38
	Gilgit	4	1.38
	Not reported/Others	4	1.38
Education level	No formal education	51	17.6
	Primary	11	3.8
	Secondary	92	31.8
	Post-Secondary	135	46.7
Occupation	Butchers	105	36.33
	Farmers/Livestock Keepers	99	34.25
	Multiple Professions	85	29.41

Most of the study participants were from Islamabad with 52.2% (151/289), whereas participants from Rawalpindi were 47.8% (138/289). The major ethnic group in this study was Punjabi with 82.0% (237/289) and the second group was of Pathans with 10.4% (30/289). The least ethnicities in this study were Urdu speaking with 1.7% (5/289), Sindhi 1.0% (3/289), Siraiki 0.7% (2/289), Kashmiris 1.4% (4/289), Gilgit 1.4% (4/289) and others or non-reported of 1.4% (4/289). On education, 17.6 % (51/289) of the participants had never attended any formal education, 3.8% (11/289) had stopped in primary while 31.8% (92/289) had attended secondary level education, and 46.7% (135/289) of the participants had attended post-secondary level of education (Table 1).

### Knowledge towards CE

It can be seen from collected data that the knowledge about CE was still very low in Rawalpindi and Islamabad. Out of 289 respondents, only 31.5% (91/289) had ever heard about zoonotic disease and 68.5% (198/289) were those who never heard before. CE being a zoonotic disease, people had little knowledge on zoonotic infections, thus, according to the survey, only 4.2% (12/289) knew about CE only, and 95.8% (277/289) had no knowledge and they never even heard about CE (Table 2).

**Table 2:** Knowledge towards cystic echinococcosis of participants in different people in study areas

Variable	Characteristics	Response									
		heard about zoonosis		heard about CE		seen hydatid disease in animal organ		seen hydatid disease in man		aware of the threats of contaminated food	
		Yes (%)	No (%)	Yes (%)	No (%)	Yes (%)	No (%)	Yes (%)	No (%)	Yes (%)	No (%)
Gender	Male	80 (30.65)	181 (69.34)	11 (4.21)	250 (95.78)	23 (8.81)	238 (91.18)	5 (1.91)	256 (98.08)	137 (52.49)	124 (47.50)
	Female	11 (39.28)	17 (60.71)	1 (3.57)	27 (96.42)	1 (3.57)	27 (96.42)	0 (0.00)	28 (100)	11 (39.28)	17 (60.71)
Statistics		*χ*^2^ = 0.51 *P* < 0.47		*χ*^2^ = 1.99 e-28 *P* < 1.00		*χ*^2^ = 0.35 *P* < 0.552		*χ*^2^ = 1.46 *P* < 0.48		*χ*^2^ = 1.27 *P* < 0.258	
Age	15 – 35	53 (32.31)	111 (67.68)	5 (3.04)	159 (96.95)	10 (6.09)	154 (93.90)	3 (1.82)	161 (98.17)	92 (56.09)	72 (43.90)
	36 – 56	32 (30.47)	73 (69.52)	7 (6.66)	98 (93.33)	13 (12.38)	92 (87.61)	2 (1.90)	103 (98.09)	48 (45.71)	57 (54.28)
	57 – 77	6 (30.00)	14 (70.00)	0 (0.00)	20 (100.00)	1 (5.00)	19 (95.00)	0 (0.00)	20 (100.00)	07 (35.00)	13 (65.00)
Statistics		*χ*^2^ = 0.09 *P* < 0.952		*χ*^2^ = 3.02 *P* < 0.21		*χ*^2^ = 3.73 *P* < 0.155		*χ*^2^ = 3.11 *P* < 0.54		*χ*^2^ = 4.7 *P* < 0.09	
Ethnicity	Punjabi	74 (31.22)	163 (68.77)	9 (3.79)	228 (96.20)	18 (7.59)	219 (92.40)	5 (2.11)	232 (97.89)	114 (48.10)	123 (51.89)
	Pathan	9 (30.00)	21 (70.00)	1 (3.33)	29 (96.66)	27 (90.00)	3 (10.00)	0 (0.00)	30 (100.00)	15 (50.00)	15 (50.00)
	Others	7 (31.81)	15 (68.18)	1 (4.54)	21 (95.45)	02 (9.09)	20 (90.90)	0 (0.00)	22 (100.00)	17 (77.27)	05 (22.72)
Statistics		*χ*^2^ = 0.02 *P* < 0.98		*χ*^2^ = 0.05 *P* < 0.97		*χ*^2^ = 133.68 *P* < 0.00001		*χ*^2^ = 2.98 *P* < 0.81		*χ*^2^ = 6.85 *P* < 0.03	
Education level	No formal education	18 (35.29)	33 (64.70)	3 (5.88)	48 (94.11)	12 (23.52)	39 (76.47)	02 (3.92)	49 (96.07)	28 (54.90)	23 (45.09)
	Primary	02 (18.18)	09 (81.81)	1 (9.09)	10 (90.90)	2 (18.18)	9 (81.81)	01 (9.09)	10 (90.90)	05 (45.45)	06 (54.54)
	Secondary	20 (21.73)	72 (78.26)	1 (1.08)	91 (98.91)	01 (1.08)	91 (98.91)	00 (0.00)	92 (100)	09 (9.78)	83 (90.21)
	Post-Secondary	41 (30.37)	94 (69.62)	7 (5.18)	128 (94.81)	08 (5.92)	127 (94.07)	02 (1.48)	133 (98.51)	106 (78.51)	29 (21.48)
Statistics		*χ*^2^ = 4.03 *P* < 0.25		*χ*^2^ = 3.59 *P* < 0.309		*χ*^2^ = 25.14 *P* < 0.00001		*χ*^2^ = 6.61 *P* < 0.085		*χ*^2^ = 103.91 *P* < 0.00001	
Occupation	Butchers	34 (32.38)	71 (67.61)	8 (7.61)	97 (92.38)	9 (8.57)	96 (91.42)	4 (3.80)	101 (96.19)	63 (60.00)	42 (40.00)
	Farmers/Livestock Keepers	35 (35.35)	64 (64.64)	0 (0.00)	99 (100.00)	11 (11.11)	88 (88.88)	0 (0.00)	99 (100.00)	26 (26.26)	73 (73.73)
	Multiple Professions	22 (25.88)	63 (74.11)	4 (4.71)	81 (95.30)	4 (4.71)	81 (95.29)	1 (1.17)	84 (98.82)	59 (69.41)	26 (30.58)
Statistics		*χ*^2^ = 1.96 *P* < 0.37		*χ*^2^ = 7.52 *P* < 0.02		*χ*^2^ = 2.47 *P* < 0.28		*χ*^2^ = 19.34 *P* < 0.0006		*χ*^2^ = 39.18 *P* < 3.1e-9	

Participants who mentioned that they had seen hydatid cysts in animal organs were with 8.3% (24/289), and 91.7% (265/289) had no knowledge and they had never seen any hydatid cysts in any organ of the animals. Out of 8.3% participants, only 1.7% (5/289) mentioned hydatid disease in man and other 98.3% (284/289) had not mentioned any case of CE. Participants were aware of the danger of eating food contaminated by dog feces with 51.2% (148/289) but none of them mentioned CE as one of the dangers, whereas 48.8% (141/289) of study participants were not aware of any threat of eating food contaminated by dog feces (Table 2).

### Attitude towards CE

Data were collected about the attitudes for CE from 289 respondents. Out of 289 respondents, only 177 (61.2%) participants had a positive response that they were at the risk of CE and 168 (58.5%) thought that people can get infected with CE from close association with dogs. Similarly, 1.7% (5/289) thought about association with people infected with CE (Table 3).

**Table 3:** Attitude towards cystic echinococcosis in different population

Variable	Characteristics	Response					
		At risk of CE		Infected with CE from close association with dogs		Association with infected people	
		Yes (%)	No (%)	Yes (%)	No (%)	Yes (%)	No (%)
Gender	Male	175 (67.04)	86 (32.95)	148 (56.70)	113 (43.29)	03 (1.14)	258 (98.85)
	Female	2 (7.14)	26 (92.85)	20 (71.42)	08 (28.57)	02 (7.14)	26 (92.85)
Statistics		*χ*^2^ = 38.23 *P* < 0.00001		*χ*^2^ = 2.25 *P* < 0.13		*χ*^2^ = 5.34 *P* < 0.02	
Age	15 – 35	108 (65.85)	56 (34.14)	128 (78.04)	36 (21.95)	88 (53.65)	76 (46.34)
	36 – 56	77 (73.33)	28 (26.66)	78 (74.28)	27 (25.71)	47 (44.76)	58 (55.23)
	57 – 77	04 (20.00)	16 (80.00)	03 (15.00)	17 (85.00)	04 (20.00)	16 (80.00)
Statistics		*χ*^2^ = 21.15 *P* < 0.000026		*χ*^2^ = 35.71 *P* < 0.00001		*χ*^2^ = 8.82 *P* < 0.01	
Ethnicity	Punjabi	144 (60.75)	93 (39.24)	159 (67.08)	78 (32.91)	177 (74.68)	60 (25.31)
	Pathan	22 (73.33)	08 (26.66)	18 (60.00)	12 (40.00)	24 (80.00)	06 (20.00)
	Others	11 (50.00)	11 (50.00)	09 (40.90)	13 (59.09)	13 (59.09)	09 (40.90)
Statistics		*χ*^2^ = 3.14 *P* < 0.21		*χ*^2^ = 6.29 *P* < 0.04		*χ*^2^ = 3.16 *P* < 0.20	
Education level	No formal education	42 (82.35)	09 (17.64)	47 (92.15)	04 (7.84)	42 (82.35)	09 (17.64)
	Primary	08 (72.72)	03 (27.27)	09 (81.81)	02 (18.18)	06 (54.54)	05 (45.45)
	Secondary	77 (83.69)	15 (16.30)	82 (89.13)	10 (10.86)	86 (93.47)	06 (6.52)
	Post-Secondary	116 (85.92)	19 (14.07)	109 (80.74)	26 (19.25)	131 (97.03)	04 (2.96)
Statistics		*χ*^2^ = 1.52 *P* < 0.67		*χ*^2^ = 5.37 *P* < 0.146		*χ*^2^ = 31.22 *P* < 0.00001	
Occupation	Butchers	89 (84.76)	16 (15.23)	97 (92.38)	08 (7.61)	94 (89.52)	11 (10.47)
	Farmers/Livestock Keepers	57 (57.57)	42 (42.42)	78 (78.78)	21 (21.21)	88 (88.88)	11 (11.11)
	Multiple Professions	74 (87.05)	11 (12.94)	69 (81.17)	16 (18.82)	78 (91.76)	07 (8.23)
Statistics		*χ*^2^ = 28.64 *P* < 0.00001		*χ*^2^ = 8.13 *P* < 0.0171		*χ*^2^ = 0.45 *P* < 0.796	

### Practices of dog owners in urban and rural areas

Out of 289 respondents, only 184 participants owned dogs and remaining 105 participants were butchers that only mentioned presence or absence of stray dogs. The numbers of respondents that responded positively about stray dogs were 51.4% (54/105), whereas 48.6% (51/105) did not responded positively. From a total of 184 dog owners, 77.2% (142/184) reported about the presence of stray dogs around their residence, whereas 22.8% (42/184) of respondents mentioned the absence of stray dogs in their area. Furthermore, participants that showed positive response regarding practices including deworming of dogs were 68.4% (126/184), received veterinary care when ill were 80.4% (148/184), family associated with dogs were 53.3% (98/184), dogs feces properly disposed-off were 52.7% (97/184), water boiling were 41.8% (77/184), hand washing when handling food were 76.6% (141/184), feeding of dogs with uncooked organs were 63.0% (116/184) and home slaughtering of animals were 48.9% (90/184). The majority of the participants revealed that they never seen inspectors when they bought meat; they admitted that they were eating uninspected meat. On hand washing, the participants reported that they washed their hands especially when they were going to eat foods, but most of the people reported that they rarely washed their hands after handling of animals. On water boiling, the participants gave several reasons why they did not boil water; some of them answered that well water were natural reservoirs of water, so, there was no need of boiling that water (Table 4), and this among butchers as well (Table 5).

**Table 4:** Practices associated with the dog owners in cities and villages in different population

Variable	Characteristics	Response									
		Feeding of dogs uncooked organs from animals		Dogs feces disposed-off		Water boiling		Hand washing		Home slaughtering of animals	
		Yes (%)	No (%)	Yes (%)	No (%)	Yes (%)	No (%)	Yes (%)	No (%)	Yes (%)	No (%)
Gender	Male	129 (49.42)	132 (50.57)	174 (66.66)	87 (33.33)	19 (7.27)	242 (92.72)	251 (96.16)	10 (3.83)	55 (21.07)	206 (78.92)
	Female	20 (71.42)	8 (28.57)	18 (64.28)	10 (35.71)	2 (7.14)	26 (92.85)	27 (96.42)	1 (3.57)	3 (10.71)	25 (89.28)
Statistics		*χ*^2^ = 4.90 *P* < 0.02000		*χ*^2^ = 0.0018 *P* < 0.96		*χ*^2^ = 0.0007 *P* < 0.97		*χ*^2^ = 1.78e-28 *P* < 1		*χ*^2^ = 1.69 *P* < 0.19	
Age	15 – 35	94 (57.31)	70 (42.68)	109 (66.46)	55 (33.53)	9 (5.48)	155 (94.51)	157 (95.73)	7 (4.26)	25 (15.24)	139 (84.75)
	36 – 56	43 (40.95)	62 (59.04)	69 (65.71)	36 (34.28)	11 (10.47)	94 (89.52)	100 (95.23)	5 (4.76)	30 (23.57)	75 (71.42)
	57 – 77	11 (55.00)	9 (45.00)	13 (65.00)	7 (35.00)	1 (5.00)	19 (95.00)	20 (100.00)	0 (0.00)	3 (15.00)	17 (85.00)
Statistics		*χ*^2^ = 6.98 *P* < 0.03		*χ*^2^ = 0.02 *P* < 0.99		*χ*^2^ = 2.52 *P* < 0.28		*χ*^2^ = 0.88 *P* < 0.64		*χ*^2^ = 7.43 *P* < 0.02	
Ethnicity	Punjabi	113 (47.67)	124 (52.32)	17 (7.17)	220 (92.82)	19 (8.01)	218 (91.98)	230 (97.04)	7 (2.95)	51 (21.51)	186 (78.48)
	Pathan	21 (70.00)	9 (30.00)	22 (73.33)	8 (26.66)	2 (6.66)	28 (93.33)	29 (96.66)	1 (3.33)	7 (23.33)	23 (76.66)
	Others	13 (59.09)	9 (40.90)	17 (77.27)	5 (22.72)	0 (0.00)	22 (100.00)	17 (77.27)	5 (22.72)	0 (0.00)	22 (100.00)
Statistics		*χ*^2^ = 5.95 *P* < 0.05		*χ*^2^ = 125.70 *P* < 0.00001		*χ*^2^ = 15.8 *P* < 0.014		*χ*^2^ = 18.42 *P* < 0.0001		*χ*^2^ = 17.5 *P* < 0.007	
Education level	No formal education	42 (82.35)	9 (17.64)	18 (35.29)	33 (64.70)	11 (21.56)	40 (78.43)	46 (90.19)	05 (9.80)	49 (96.07)	2 (3.92)
	Primary	7 (63.63)	4 (36.36)	4 (36.36)	7 (63.63)	02 (18.18)	9 (81.81)	10 (90.90)	01 (9.09)	6 (54.54)	5 (45.45)
	Secondary	42 (45.65)	50 (54.34)	28 (30.43)	64 (69.56)	30 (32.60)	62 (67.39)	90 (97.82)	02 (2.17)	24 (26.08)	68 (73.91)
	Post-Secondary	72 (53.33)	63 (46.66)	99 (73.33)	36 (26.66)	84 (62.22)	51 (37.77)	135 (100.00)	00 (0.00)	12 (8.88)	123 (91.11)
Statistics		*χ*^2^ = 19.0419 *P* < 0.0002		*χ*^2^ = 48.48 *P* < 0.00001		*χ*^2^ = 36.43 *P* < 0.00001		*χ*^2^ = 14.97 *P* < 0.00183		*χ*^2^ = 134.54 *P* < 0.00001	
Occupation	Butchers	33 (31.42)	72 (68.57)	95 (90.47)	10 (9.52)	0 (0.00)	105 (100.00)	102 (97.14)	3 (2.85)	0 (0.00)	105 (100.0)
	Farmers/Livestock Keepers	57 (57.57)	42 (42.42)	33 (33.33)	66 (66.66)	21 (21.21)	78 (78.78)	96 (96.96)	3 (3.03)	58 (58.58)	41 (41.41)
	Multiple Professions	59 (69.41)	26 (30.58)	64 (75.29)	21 (24.70)	0 (0.00)	85 (100)	80 (94.11)	5 (5.88)	0 (0.00)	85 (100.0)
Statistics		*χ*^2^ = 29.31 *P* < 0.00001		*χ*^2^ = 78.85 *P* < 3.2e-16		*χ*^2^ = 324.17 *P* < 2.2e-16		*χ*^2^ = 1.42 *P* < 0.49		*χ*^2^ = 410.7 *P* < 2.2e-16

**Table 5:** Compareness the knowledge and awareness/practice related to CE in the Butchers in the study areas

Response	No of people reported about stray dogs	Ever give meat to dogs	Type of meat			Any specific organ	No. of people give organs to eat	Waste Disposal	No. of people dispose in	Awareness regarding eating contaminated food
			Healthy	Unhealthy	None					
Yes	54	33	2	34	69	Kidney	1	Bins	69	63
No	51	72				Liver	2	Gutters	3	42
						Lungs	5	Sold	23	
						None	37	Water	3	
						Others	15	Other	7	

### Statistical analysis for knowledge and practices towards CE

The statistical analysis showed the determinants of knowledge and attitudes towards CE and found out the most important factors associated with CE.

#### Practices associated with knowledge towards CE

The factors that determined knowledge about CE included age, presence of inspectors at the source of meat, slaughtering animals at home, dog ownership, animal management system, education level, religion and perception about the disease. The statistical analysis showed that only data collected from villagers, compared between butchers and farmers about knowledge regarding being aware of eating contaminated food with dogs feces was significantly different (*P* < 0.05) with respect to responses in the studied groups. All other questions on the subject of knowledge such as ever heard about CE (*P* = 0.603), ever been heard about zoonosis (*P* = 0.663), ever seen hydatid cyst in animal (*P* = 0.542) and ever seen hydatid cyst in man (*P* =0.459) showed highly non-significant difference (*P* > 0.05). The results showed that only the butchers were aware of the knowledge of eating contaminated food but farmers were not aware of eating contaminated food with dog feces, while both butchers and farmers were not aware about the knowledge on zoonosis, CE, ever seen hydatid cyst in animal and human. It means that due to lacks of knowledge they were at higher risk of infection with cystic echinococcosis (Table 6).

**Table 6:** Differences on knowledge about CE in different occupation (butchers/ farmers) in the study areas

S. No.	Knowledge	Occupation	No. of peoples responded		*χ* ^2^	*OR*	Confidence Interval (95%)	*P* value
			Yes (%)	No (%)				
1	Ever heard about Zoonoses	Butchers	34 (32.38)	71 (67.61)	0.09	1.14	0.614 – 2.12	0.663
		Farmers	35 (35.35)	64 (64.64)				
2	Ever heard about C.E	Butchers	8 (7.61)	97 (92.38)	0.27	1.670	0.485 – 5.745	0.603
		Farmers	0 (0.00)	99 (100.00)				
3	Ever seen hydatid C.E in animal	Butchers	9 (8.57)	96 (91.42)	128.6	0.0117	0.004 – 0.029	0.542
		Farmers	11 (11.11)	88 (88.88)				
4	Ever seen hydatid cyst in man	Butchers	4 (3.80)	101 (96.19)	1.20	2.711	0.277 – 26.554	0.459
		Farmers	0 (0.00)	99 (100.00)				
5	Aware of eating contaminated food with dogs faeces	Butchers	63 (60.00)	42 (40.00)	22.23	0.239	0.125 – 0.447	1.343e-06
		Farmers	26 (26.26)	73 (73.73)				

#### Practices associated with attitude towards CE

Practices/attitude were determined from several factors which included age, occupation, tribe, religion tribe, gender, and close association with dogs; feeding of dogs uncooked organs from animals, dog’s faeces properly disposed-off, water boiling, hand washing and home slaughtering of animals.

We compared the relation between practices and attitudes. “Bad practice” included presence of stray dogs, feeding of dogs with uncooked organs from animals and home slaughtering of animals. “Proper practice” included deworming of dogs, received veterinary care when sick, dogs feces properly disposed-off, water boiling and hand washing. The statistical analysis showed that there was highly significant difference (*P* < 0.05) among all practices associated with exposure to the factors e.g. presence of stray dogs, feeding of dogs with uncooked organs from animals, home slaughtering of animals and other proper practices including deworming of dogs, received veterinary care when sick, dogs feces properly disposed-off, water boiling and hand washing. Only practice i.e. family associated with dogs showed no significant difference (*P* > 0.05). Here, we performed 2X1Chi square test, in order to check whether there was some significant difference between affirmative and negative responses for each of the binomial variable within villager’s group (presence of stray dogs, deworming of dogs, etc.). Since people were not following cleanliness and hygiene, they may be at risk of disease. The term significance was used to explain that the people were aware about the practices that may lead to CE (Table 7).

**Table 7:** Statistical Analysis for the Practices of villagers that may lead to CE

S. No	Practices	No. of rural peoples responded (*n* = 99)		*χ* ^2^	*P value*
		Yes (%)	No (%)		
1	Presence of stray dogs	70 (70.70)	29 (29.29)	16.98	3.778e-05
2	Deworming of Dogs	46 (46.46)	53 (53.53)	0.49	0.4817
3	Received veterinary care when sick	72 (72.72)	27 (27.27)	20.45	6.106e-06
4	Family associated with dogs	49 (49.49)	50 (50.50)	0.01	0.919
5	Feeding of dogs uncooked organs from animals	57 (57.57)	42 (42.42)	2.2	0.13
6	Dogs feces properly disposed-off	33 (33.33)	66 (66.66)	11	0.0009
7	Water boiling	21 (21.21)	78 (78.78)	32.8	1.012e-08
8	Hand washing	96 (96.97)	3 (3.03)	87.36	2.2e-16
9	Home slaughtering of animals	58 (58.58)	41 (41.41)	2.91	0.087

## Discussion

Pakistan is a country with a low socio-economic status. It is highly populated with approximately 200 million inhabitants, with most of these living in rural areas or very crowded urban areas with poor sanitary facilities. A large proportion of Pakistanis is affiliated with agriculture and local dairy farming on a small scale. The workers on these small farms come into close contact with animals and since proper health and hygiene principles are not strictly followed, thus, the inhabitants of these areas are also at high risk of acquiring *Echinococcus* spp*.* infections [11]. Humans can become infected through ingestion of parasite eggs in contaminated food, water or soil, or via direct contact with animal hosts. It has been shown that common sheep (G1) and buffalo (G3) strains of *E. granulosus* are circulating among livestock in Punjab and that these strains are highly adaptable to goats, camels and cattle. The molecular characterization of human cysts infected with *Echinococcus* spp. belonged to common sheep strain (G1) of *E. granulosus*, reinforcing the fact that this strain has potential for zoonotic transfer. Both morphological and molecular characterisation of *Echinococcus* spp. in Pakistan support findings similar from other parts of the world, suggesting that *Echinococcus* spp. of sheep and buffalo origin is phenotypically and genetically similar as worldwide. This adds further evidence to support its recognition as one species, namely *E. granulosus sensu stricto* [9, 10]. Some of the researchers conducted some surveys in China [12], Morocco [13], Algeria [14], Peru [15] and Uganda [16] based on Knowledge, attitude, practices and risk factors analysis.

### Socio-demographic factors

The socio-demographic factors indicating ethnicity, age, education, occupation and nationality were included in the survey. These factors had very important impact on zoonotic transmission of echinococcosis in Pakistan. In these ethnic groups, poor hygienic conditions are more common. The presence of different ethnic groups shows multicultural interactions that may contribute to the complexity of CE.

Similar observations were reported in Kasese district of Uganda where interviewed people were adults. This was because it was the intention of their study to interview only adult individuals as the inclusion criteria [9]. Adults were more likely to be knowledgeable about CE since it is a chronic disease. However this relationship was not significant with their p-value (*P* <0.63) and the fact that disease was most prevalent in adults of 20 years and above [17]. Age contributed to the overall models that determined knowledge and perception towards CE. There was no significant association between tribe and knowledge and attitude with a *P* > 0.73. This was contrary to what other authors have found such as Schantz et al. [18], who reported CE to be more popular in American Indian, Zuni and Santo tribes in New Mexico.

However, all dog owners, people with livestock including dog and butchers were included in the present study. All participants that were 15 years of age and above were included in this study. Children less than 15 years were excluded from this study, as well as houses with animals but no dogs. The participants that showed positive response in our study regarding practices included deworming of dogs, 68.5% (126/184). Tribes were also significantly associated with other factors. For example, those who were more likely to feed uncooked meat to dogs, they were less likely to wash their hand and boil water for domestic use. The variable of tribe also played a significant role in models that determine knowledge, attitude, and practices towards CE. The reason behind tribe playing critical role in determining these factors was that different tribes had different cultural practices that acted as social determinants of the disease. CE is one of the diseases that are socially constructed due to different cultural practices in different tribes like keeping many dogs, keeping a lot of livestock and a culture of preserving stray dogs reported in China [19].

Another socio-demographic factor that was measured was religion; all of the study participants were Muslims 100%, (*n* = 289). This was significant because we know that religious virtues can determine some practices like most of the Islamic communities do not have a common habit of keeping dogs unlike the Christian values. In addition, different religions have different slaughter habits that can influence the behaviour of feeding dogs with cysts. As it was observed in strong Tibetan tribes such as Sichuan kept many guard dogs where as strong Buddhists beliefs did not support the elimination of stray dogs [19], such factors have been shown to contribute to prevalence of CE. Religion was statistically significantly with many variables in study such as knowledge and perception about CE. Muslims were more knowledgeable about CE (*OR* = 5.6) than other religions. This was consistent with what Youngster in 2002 reported in Muslim communities in Southern Israel where the prevalence of CE was found to be very high because of their slaughtering habits [9].

Occupation of an individual has been found to be one of the most important factors in the epidemiology of CE disease. The present study showed that, butchers and farmers associated with dogs and livestock were at high risk to get infected with CE. From the study in Uganda Kasese district, out of the 384 participants, 54.6% were pastoralists, 22.5% were peasants, there was only one hunter, and other occupations accounted for only 15% [9]. Pastoralists were the majority because this study specifically targeted pastoral communities where the incidence of CE had been documented by many authors to be high [20–22]. Hence, occupational activity contributed significantly to determining other behaviors such as attitude and perception, hand washing, water boiling and in the models that determined these practices. Peasants for example had been found to be at high risk of developing the disease as shown in the Xiji County of China [23]. Although CE had been reported in hunters [24], there was no significant numbers of hunters surveyed in this study.

Livestock had been reported as a major risk factor for CE in multiple other studies in China [25, 26], Tunisia [27] and Peru [28]. However, it should be emphasized that whilst disease distribution was closely linked to zones where livestock was kept, some authors reported that there was no correlation between animal density and disease frequency [29].

Among 289 surveyed individuals, 17.6% (51/289) of the participants had never attended any formal education, 3.8% (11/289) had stopped in primary, while 31.8% (92/289) had attended secondary level education and the 46.7% (135/289) of the participants had attended the post-secondary level of education. CE being a zoonotic disease, people had little knowledge on zoonotic infections. Thus, according to the survey, only 4.2% (12/289) knew about CE only, and 95.8% (277/289) had no knowledge and they never even heard about CE. This was an indicator of low literacy levels. Although education was important in knowledge acquisition, it was not significantly associated with knowledge about CE. When comparing among individuals with different educational background, it has been showed that knowledge about the disease was same irrespective of the level of education of individuals. There was no significant difference between different education levels as far as knowledge about CE was concerned. This was more likely scenario with neglected tropical disease like CE, even the educated communities did not usually know about it. However, education played a critical role determining other factors associated with CE such as perceptions about the disease, deworming of dogs, hand washing, water boiling, feeding dog infected cysts, etc. This showed a critical role of education in controlling the transmission of CE. If education levels are high, there are chances that the transmission level of the disease will be low. The role of education in reducing or controlling CE has been dully emphasized by Ozcelik at al. [30], who designed health education messages to control the disease in Turkey and also in Sardinia [31]. Similarly, the socio-economic situation has been reported as a potential risk factor for CE transmission in China [25, 26].

### Knowledge about CE

It can be seen from the collected data that the knowledge about CE was still very low in Rawalpindi and Islamabad. Out of 289 respondents, only 31.5% (91/289) had ever heard the zoonosis. Given that it was being a zoonotic disease, people had little knowledge on zoonotic infections; so, according to the survey only 4.2% (12/289) know about CE only. Participants were aware of the threat of eating contaminated food by dog feces with 51.2% (148/289), but none of them mentioned CE as one of the threat. It might be due to the low level of knowledge about this disease, especially attributed to poor diagnosis as reported by medical and veterinary physician. The disease is expensive to diagnose and treat, and time is consumed in alternative treatment method by going to traditional healers. Knowledge about the disease is very important if its prevention and control strategies are to be effective. With these low levels of knowledge of the disease, it  meant the population in Rawalpindi and Islamabad is unaware of the factors responsible for developing the disease.

Many authors [15, 32] have emphasized the importance of knowledge and education about the CE in instituting control and prevention strategies. It was found that knowledge about CE was determined by many factors, which include age, presence of inspectors at the source of meat, slaughtering animals at home, dog ownership, animal management system, education level, religion, and perception about the disease [33]. This was consistent with findings in Jordan [34]. Therefore, to improve on knowledge about the disease, the above factors need to be modified.

### Attitude towards CE

Perception about a disease can influence its epidemiology. If people perceive themselves at risk of acquiring the disease, they are more likely to guard against getting the disease and vice versa. Data were collected about the attitudes for CE from 289 respondents. Out of 289 respondents, only 177 (61.2%) participants had a positive response that they were at the risk of CE and 168 (58.1%) think that they can get infected with CE from close association with dogs. Similarly, 1.7% (5/289) thought that association with people infected with CE can increase the risk of CE infection. From a total of 184 dog owners, 77.2% (142/184) reported about the presences of stray dogs around their residence and they were not thinking that at risk of being infected by CE, whereas 77.2% thought that they can get hydatidosis from close association with dogs. This one still showed lack of sensitization on the threats of CE in pastoral communities of Rawalpindi and Islamabad. Therefore, people’s attitudes/behaviour should be changed to influence disease dynamics. The other factors affecting the behaviour towards CE included lack of deworming of dogs as 31.5% (58/184), not received veterinary care during illness phase, family associated with dogs as 53.3% (98/184), feeding dogs with uncooked organs of animals as 63.0% (116/184), dog faeces not properly disposed-off as 47.32% (87/184), no water boiling as 58.2% (107/184), no hand washing when handling food as 23.4% (43/184) and home slaughtering (48.9%). The rest of the factors were least significant, which showed that their presence or absence did not affect CE development when tested with perception about the disease. These findings corroborated those reported by Azlaf and Dakkak [35] in other regions of Morocco, as well as Benabid et al. [27] in Tunisia. Similarly, a study conducted in Eastern Algeria found that 91.1 % of rural households did not deworm their dogs, although the percentage of households feeding dogs infected liver and lungs was found to be lower at 12.1% [14].

This was also documented by Hemachander at al*.* [36] who found out that people knew only rabies as a disease they can acquire from dogs. According to this study, education was key determinant of perception about the disease, and also, people had no idea of zoonoses from dogs such as CE. Therefore, people attitudes should be changed to influence disease dynamics. According to one study, education was key determinant of perception about the disease, however other factors that influenced attitude towards CE include people who had seen the disease (*OR* = 3.82), those who have knowledge about zoonoses (*OR* = 4.7) and home slaughtering (*OR* = 0.3) in Peru [15].

### Practices associated with CE

Several practices were associated with CE. In surveyed area of Rawalpindi and Islamabad, where stray dogs exists, they were at risk of developing CE. This was because of poor deworming habit of dogs where only 68.4% of 184 participants dewormed their dogs. This means that dogs remain as carriers of the adult worm, hence leading contamination of the environment with fecal material. The close association of people with dogs 53.2% (98/184) especially children were more at risk and they can acquire this disease when they were still young and signs come later in life further exacerbates this factor. Deworming had been shown to significantly reduce the spread of disease both in dogs and man. It has been showed that dog ownership was a risk factor for CE [23, 37, 38]. Many areas where CE has been diagnosed also have high levels of stray dogs [39] and found 50% prevalence in stray dogs in Iraq [40].

As we found in our study, a high population of stray dogs (77%) reported was contributing to the development of CE. Among 184 sample size, 31.5% were practicing home slaughtering. With this practice, there was  no meat inspection. Home slaughtering has been documented as one of the risk factors for CE [41, 42] and was found highly associated with the prevalence of CE. Similarily abattories especially in rural areas, were considered as the main source of dog infection in Morocco, namely in Rabat [43], in Quarzazate and in Tetouan [44, 45]. A model was built to find out which factors when combined together determine this behaviour of home slaughtering and this included tribe, religion, gender, occupation, animals kept, dog ownership, daily practices and attitude towards CE. Hygiene and sanitation status can influence the epidemiology of a disease. Poor hygiene practices such as poor hand washing habits, drinking un-boiled water and eating contaminated food can lead to risk of developing CE [15].

In our study the hygiene levels were low where by only 76.6% (*n=184*) washed their hand after handling dog. On investigating the factors associated with hand washing, hand washing was positively associated with several factors. All these factors were statistically significant meaning that to improve on this behaviour, we need to modify these factors, and in the process, we are able to reduce the risk of transmission of the disease between different animals and man. A model for factors that determine hand washing include tribe, religion, sex, age, education level, animals kept animal management system, knowledge of CE and knowledge about zoonoses. Almost the same factors play a critical role in determining the practice of water boiling which was 7.3% in our study and 92.7% who did not use boil water. Similar observations were also reported previously [13].

Due to the frequent outbreaks in different parts of Pakistan in the recent past, echinococcosis is being described as a neglected tropical disease and is considered one of the most neglected parasitic diseases in the country. CE in Pakistan is a serious burden disproportionally borne by poor, rural and livestock keeping communities. CE has a worldwide geographical distribution with endemic foci on every inhabited continent. In endemic regions, predominantly settings with limited resources, there are high numbers of echinococcosis patients, as these communities do not have access to appropriate treatment. In Pakistan, there are limited reports on echinococcosis. The disease is prevalent in human and livestock, but this has not been sufficiently explored yet. Pakistan is an agricultural country and due to the disease’s zoonotic mode of transmission, there is a dire need of future research on CE based on molecular basis [11].

The present paper is an effort to highlight the importance of difference among knowledge, attitude and practices of individuals of endemic area that facilitate the zoonotic transmission of CE in Pakistan. The present study highlights the home slaughtering, stray dogs, poor hygienic practices, close association of dog owners, feeding of stray dogs with lungs and liver from butcher shops, no knowledge about CE, use of untreated water without boiling were the contributing factors for zoonotic transmission of CE in Pakistan.

Active surveillance of CE has been undertaken in Pakistan since there is a dire need of establishment of the One Health platform by the Ministry of Health. In rural areas at the provincial level, the Committee should be responsible for the awareness, application of control measures and for monitoring the evolution of disease, but this is heavily focused on human disease. Animal cases should be detected during routine abattoir inspection. This work has identified the drivers for disease transmission and sets a clear agenda and priorities for controlling the disease in Rawalpindi/Islamabad. Unfortunately the Pakistan has the lack of platform to roll-out inter-sectoral and integrated control strategies, now it is time for political will to follow and for the Interministerial Committee to be given the means and resources to put this knowledge into practical use.

## Conclusion

It was concluded from this study that the knowledge about CE was generally very low. Just like many other neglected zoonoses, the residents of Rawalpindi/Islamabad did not know the threats associated dogs infected with *E. granulosus* and cattle with hydatid cysts, its life cycle, control and preventive measures. There were many practices and factors that can predispose the population of twin cities to infection by CE or people could actually be infected by the disease since some lesions/cysts were found in animals as well as in humans. It should be necessary to create awareness among people and other areas of Pakistan should also be explored. The emerging trend of echinococcosis in Pakistan brings the disease to limelight for future research. In order to control the disease, complete surveillance should be done which in turn weighs down the disease progress.

## Additional file

Additional file 1: Multilingual abstracts in the six official working languages of the United nations. (PDF 357 kb)
