# Supplementary material for: Knowledge, attitudes and practices related to cystic echinococcosis endemicity in Pakistan
Source: Infect Dis Poverty. 2018 Jan 22;7:4. doi: 10.1186/s40249-017-0383-2 (PMC5776779; doi:10.1186/s40249-017-0383-2)
Supplement: Additional file 1: — Multilingual abstracts in the six official working languages of the United nations. (PDF 357 kb) [file 40249_2017_383_MOESM1_ESM.pdf]

Translation of the abstract into the five official working languages of the United Nations

المعرفة والمواقف والممارسات المتعلقة بالمشوكات الكيسية المتوطنة في باكستان

Aisha Khan, Kashf Naz, Haroon Ahmed, Sami Simsek, Muhammad Sohail Afzal, Waseem Haider, Sheikh Saeed Ahmad, Sumaira Farrakh, Wu Weiping, Guan Yayi

الملخص

الخلفية: تعتبر المشوكات الكيسية مشكله صحية للإنسان والحيوان في العديد من المناطق الموبوءة في جميع انحاء العالم. ويعتبر مرضا حيوانيا مهملا ينتج عن الإصابة بيرقة الكيسة العدارية للديدان الشريطية من نوع المشوكات وهناك دراسات قليلة عن المشوكات الكيسية في باكستان.

الأساليب: أجريت دراسة استقصائية شامله لمعرفه المعارف والمواقف والممارسات الحديثة بشأن وجود إصابات بالمشوكات الكيسية بين الجزارين ومالكي الكلاب في المناطق الحضرية والريفية من روالبندي/إسلام أباد، باكستان. وقد تم تجميع البيانات الكمية في شكل استبيان لاستقصاء المعرفة والوعي بالمشوكات الكيسية لدي افراد المجتمعات المحلية وممارساتهم الروتينية التي تتسبب في إصابتهم بالكيسة العدارية وتم تقييم الممارسات والهياكل الأساسية لمحلات السلخانة او الجزارة ودورها في نقل المشوكات الكيسية.

النتائج: المشاركون في الدراسة الحالية كانوا من مالكي الكلاب والأشخاص الذين يحتفظون بالماشية حيث تم مقابله ما مجموعه ٤٠٠ شخصا وتم تجميع ٢٨٩ استبيانا وأظهرت النتائج ان ٤,١٪ فقط من الأشخاص قد سمعوا عن المرض و٥٨,١٪ كانوا مرتبطتين ارتباطا وثيقا بالكلاب وكانت ٦٣٪ من الكلاب في المنطقة تتغذي علي الأعضاء غير المطبوخة (الكبد/الرئتين) من الماشية المذبوحة في حين أن ١٠٠٪ من الكلاب عند محلات الجزارة كانت تستهلك الأعضاء غير المطبوخة وكان الذبح المنزلي شائعا في ٢٠,٠٦٪ ومن بين الجزارين كانت هناك نسبة ٣٢,٣٪ سمعوا عن الامراض الحيوانية ونسبة ٧,٦١٪ قد سمعوا عن مرض المشوكات الكيسية واظهر التحليل الإحصائي ان هناك فرقا كبيرا للغاية ($P < 0.05$) بين معظم الممارسات المرتبطة بانتشار المرض.

الاستنتاج: يستنتج من الدراسة الحالية ان المعرفة والوعي لدي سكان روالبندي/إسلام أباد كانا منخفضين وتسببت الكلاب والمعرفة السيئة عن مرض المشوكات الكيسية بين أعضاء المجتمع والجزارين في سهولة انتقال المرض ولذلك فمن الضروري تعزيز الوعي بين الناس وهناك حاجة إلى نشر المعرفة والوعي الجيد والممارسات السليمة المتعلقة بمرض المشوكات الكيسية ليس فقط في منطقة الدراسة ولكن أيضا في المناطق الأخرى من باكستان.

Translated from English version into Arabic by Mohamed Habib

与巴基斯坦囊型包虫病流行相关的知识、态度及行为调查

Aisha Khan, Kashf Naz, Haroon Ahmed, Sami Simsek, Muhammad Sohail Afzal, Waseem Haider, Sheikh Saeed Ahmad, Sumaira Farrakh, Wu Weiping, Guan Yayi

摘要

背景：囊型包虫病（CE）是由细粒棘球绦虫的幼虫（棘球蚴）引起的人畜共患病，是全球许多流行区影响人类和动物健康的重要公共卫生问题。迄今，巴基斯坦对包虫病的研究尚很有限。

方法：通过横断面调查，了解巴基斯坦拉瓦尔品第（Rawalpindi）/伊斯兰堡（Islamabad）地区城市和农村地区屠夫和养犬者有关囊型包虫病发生的知识、态度和行为。采用问卷调查方法，收集定量资料，了解社区成员对 CE 的认识、意识和常规行为。本研究还评估了屠宰场/肉店的操作和基础设施以及它们在囊型包虫病传播中的作用。

结果：本研究涉及的参与者为养犬人和养动物的人。共有 400 人接受访谈，收到 289 份问卷。结果显示只有 4.1%的人听说过该病，58.1%的人与犬关系密切。研究地区内 63%的犬食用生的动物脏器（肝脏/肺脏），而屠夫店内的犬 100%食用未经烹煮的脏器。家庭自行屠宰动物比较普遍，占 20.06%。屠夫中有 32.3%的人听说过人畜共患病，7.61%知道 CE。统

计分析显示，存在这些行为与 CE 流行相关（P <0.05）。
结论：根据研究结果，拉瓦尔品第/伊斯兰堡人对囊型包虫病的认识和认知程度较低。这是由于社区成员和屠夫对犬和囊型包虫病知之甚少，于是造成了疾病的传播，因而，有必要加强囊型包虫病知识的宣传，提高认知，改进行为。

Translated from English version into Chinese by Ya-Yi Guan

Les connaissances, les attitudes et les pratiques liées à l'échinococcose cystique endémique au Pakistan

Aisha Khan, Kashf Naz, Haroon Ahmed, Sami Simsek, Muhammad Sohail Afzal, Waseem Haider, Sheikh Saeed Ahmad, Sumaira Farrakh, Wu Weiping, Guan Yayi

Résumé
Contexte: L'échinococcose cystique (EC) est un problème de santé humaine et animale dans de nombreuses régions endémiques du monde entier. Elle est considérée comme une maladie zoonotique négligée causée par la larve (hydatide) d'échinococcus granulosus. Il existe peu d'études sur l'échinococcose au Pakistan.
Méthodes: Une enquête transversale a été menée pour connaître les connaissances, attitudes et pratiques récentes sur l'apparition de l'échinococcose cystique chez les propriétaires de boucherie et de chiens dans les zones urbaines et rurales des régions de Rawalpindi / Islamabad, au Pakistan. Les données quantitatives ont été recueillies sous forme de questionnaire pour enquêter sur les connaissances et la conscience de l'EC chez les membres de la communauté et leurs pratiques de routine qui sont derrière les facteurs impliqués dans l'infection de hydatide cystique. Les pratiques et l'infrastructure des abattoirs / boucheries et leur rôle dans la transmission de l'échinococcose cystique ont également été évalués dans la présente étude.
Résultats: Les participants impliqués dans cette étude étaient des propriétaires de chiens et des personnes qui élevaient des animaux. Au total, 400 personnes ont été interrogées et 289 questionnaires ont été reçus. Les résultats ont montré que seulement 4.1% des personnes ont entendu parler de la maladie, et 58.1% étaient étroitement associées aux chiens. 63% des chiens dans la zone d'étude consommaient des organes non cuits (foie / poumons) d'animaux abattus tandis que 100% des chiens dans les boucheries consommaient des organes non cuits. L'abattage à domicile était courant dans 20.06% des cas. Parmi les bouchers, 32.3% avaient entendu parler des zoonoses et 7.61% connaissaient l'EC. L'analyse statistique a montré qu'il existe une différence très significative (P <0.05) entre la plupart des pratiques associées à la prévalence de l'EC.
Conclusion: Il ressort de la présente étude que les connaissances et la conscience de l'EC chez les habitants de Rawalpindi / Islamabad étaient faibles. Comme les membres de la communauté et les bouchers ont une faible connaissance des chiens et de l'EC, la transmission de la maladie est facilitée. Il devrait être nécessaire de renforcer la conscience parmi les gens et de diffuser les connaissances, la bonne conscience et les bonnes pratiques liées à l'EC non seulement dans la zone d'étude, mais aussi dans d'autres régions du Pakistan.

Заинтересованные знания, отношения и практики с распространением Пакистанского кистозного эхинококкоза

Aisha Khan, Kashf Naz, Haroon Ahmed, Sami Simsek, Muhammad Sohail Afzal, Waseem Haider, Sheikh Saeed Ahmad, Sumaira Farrakh, Wu Weiping, Guan Yayi

Конспект

Фон: кистозный эхинококкоз (CE) – большая проблема о влиянии на здоровье людей и животных в многих распространенных регионах в мире. По извещению, данная болезнь – пренебрегающая совместная болезнь людей и животных из-за личинок (эхинококк) мелкозернистых эхинококков. Исследование кистозного эхинококкоза ограниченное в Пакистане.

Способы: путём расследования поперечного разреза, знают заинтересованные знания, отношения и практики с кистозным эхинококком мясников и владелец по держанию собаки в последние дни в регионах Rawalpindi / Islamabad Пакистана. Путём анкетного обследования собирают документы определенного количества, знают познание и сознание CE из членов сообщества, и постоянные практики в заинтересованных факторах с загрязнением эхинококков. В данном исследовании ещё оценивали операцию и инфраструктуры на площади бойни / в мясном магазине, инфраструктуры и действия кистозных эхинококков.

Результаты: участники в данном исследовании—владелец по держанию собаки и животных. Всего лишь 400 приняли интервью, получили 289 опросных листов. Результаты показывают то, что только 4.1% людей слышали данные болезни, 58.1% людей имеют тесное отношение с собачками. В исследовательской зоне 63% собак кушают сырые органы животных (печень/легкие), а в лавке мясника 100% собак кушают не варенные внутренние органы. 32.3% мясников слышали то, что люди заболевают данной болезной, 7.61% мясников знают CE. Статистический анализ показывает то, что в большинстве заинтересованных способов с распространением CE возникают значительные разницы ($P < 0.05$).

Вывод: В исследовательских результатах в настоящее время видно, степень по познанию кистозного эхинококкоза из людей в регионах Rawalpindi / Islamabad более низкая. Вот это члены из сообщества и мясники мало знают собаки и кистозные эхинококкозы, и это вызывало распространение болезней, поэтому, необходимо распространять и повышать сознание знаний кистозных эхинококкозов, улучшать поведения и практики.

Conocimientos, Actitudes y Prácticas Relacionados con la Epidemia de Equinococosis Quística en Pakistán

Aisha Khan, Kashf Naz, Haroon Ahmed, Sami Simsek, Muhammad Sohail Afzal, Waseem Haider, Sheikh Saeed Ahmad, Sumaira Farrakh, Wu Weiping, Guan Yayi

Extracto

Antecedentes: La equinococosis quística (CE) es un gran problema que afecta la salud humana y animal en muchas áreas endémicas del mundo. Se ha informado de que es una enfermedad zoonótica desatendida causada por larvas de Echinococcus granulosus (equinococosis). Existen estudios muy limitados sobre la equinococosis en Pakistán.

Métodos: Se ha llevado a cabo una investigación de sección transversal para conocer los conocimientos, actitudes y prácticas recientes sobre la ocurrencia de equinococosis quística de los carniceros y dueños de perros en las áreas urbanas y rurales de las regiones de Rawalpindi/Islamabad, Pakistán. Se ha adoptado una encuesta en forma de cuestionario para recoger datos cuantitativos, y conocer los conocimientos y conciencia de los miembros de comunidades sobre la CE, así como sus prácticas rutinarias en los factores relacionados con la infección de equinococosis. En dicho estudio, también se han evaluado las operaciones e infraestructuras de los mataderos/carnicerías y su papel en la transmisión de la equinococosis quística.

Resultados: Los participantes en dicho estudio eran dueños de perros y personas que tenían animales, con un total de 400 personas entrevistadas y 289 cuestionarios recibidos. Los resultados

han mostrado que sólo el 4.1% de las personas habían oído dicha enfermedad, y el 58.1% tenían una relación estrecha con los perros. El 63% de los perros en el área de estudio consumían órganos de animales crudos (hígado/pulmón), y el 100% de los perros en las carnicerías consumían órganos sin cocinar. La matanza era relativamente común en el 20.06％ de las familias. El 32.3% de los carniceros habían oído la enfermedad zoonótica, y el 7.61％ sabía la CE. El análisis estadístico ha mostrado que existe una diferencia notable ($P <0.05$) entre la mayoría de las prácticas relacionadas con la epidemia de CE.

Conclusión: Desde los resultados actuales del estudio, se puede ver que las personas de Rawalpindi/Islamabad tienen un relativamente bajo nivel de conocimiento y conciencia sobre la equinococosis quística, esto se debe a que los miembros de comunidades y carniceros conocen poco sobre los perros y equinococosis quística, resultando así en la transmisión de la enfermedad. Por eso, es necesario fortalecer la difusión de conocimientos sobre equinococosis quística, reforzar la conciencia, y mejorar los comportamientos y prácticas en Pakistán.
